# Inpatient postoperative undesirable side effects of analgesics management: a pediatric patients and parental perspective

**DOI:** 10.1097/PR9.0000000000000845

**Published:** 2020-09-01

**Authors:** Bianca Chabot, Catherine E. Ferland

**Affiliations:** a Department of Clinical Research, Shriners Hospitals for Children-Canada, Montreal, QC, Canada; b Department of Anesthesia, McGill University, Montreal, QC, Canada; c Child Health and Human Development Research Axis, Research Institute—McGill University Health Centre, Montreal, QC, Canada

**Keywords:** Pediatrics, Pain, Medication, Undesirable side effects, Perioperative pain

## Abstract

**Introduction:**

The use of analgesics for the treatment of post-operative pain is common, however, such medications are known to have potential side effects. These undesirable secondary effects can have an important impact on patients and impede their recovery.

**Objectives:**

A review of the literature was conducted in order to gain a better understanding of the challenges confronted by pediatric patients in the acute post-operative period in terms of the side effects of analgesics.

**Methods:**

An online search of keywords (pediatric, analgesic, pain medication, side effects, adverse effects, nausea and vomiting, post-operative, post-discharge, self-care, self-management, management, self-care strategies, patient expectations, patient concerns and education) using PubMed, Medline and Scopus databases, and using the snowballing method of reference tracking was conducted.

**Results:**

A total of 10 studies (N = 10, 871 participants) published between 1990 and 2019 were reviewed. Common side effects experienced by patients were nausea, vomiting, and pruritus. Patients' parents reported having many concerns about analgesic use and reported a lack of knowledge on pain medications and side-effect management.

**Conclusion:**

Areas of improvement in clinical practice include providing the patient and their parents with more information about the post-operative period, analgesic use, and side effects as well as prescribing appropriate treatments to alleviate side effects. This review reveals a lack of qualitative data on pain management and related undesired side effects in pediatric patients having undergone inpatient surgery in addition to the consequences on patients' daily living and on the self-care strategies they engage in to attenuate such effects.

## Abstract 2

There is a literature gap concerning the management of undesirable effects of analgesics prescribed to pediatric inpatients and a need to improve education provided about pain management.

## 1. Introduction

Postoperative pain in pediatric patients has been associated with limitations in daily living such as reduced school attendance, extracurricular activities, social activities, household tasks, and sleep quality.^8^ Not only can these consequences have a severe impact on the patient, but they also place a burden on their families, their social environment and the healthcare system at large.^7^ It is, therefore, critical to manage and treat postoperative pain as well as possible. However, even if the best pain management is provided and pain is well controlled, recovery may be prolonged due to the side effects of pain medication.^13^ The use of analgesics such as opioids, acetaminophen, and nonsteroidal anti-inflammatory drugs for the treatment of postoperative pain is common in both adult^1,2,16,29^ and pediatric patients.^2^ However, such medications are known in both populations for common potential side effects such as sedation, nausea, vomiting, constipation, itching, dizziness, headache, respiratory depression, and digestive organ irritability.^2,23,29^ A better understanding of the challenges confronted by pediatric patients in the acute postoperative period in terms of analgesic-caused side effects may present valuable details to improve care provided.

A review of the scientific literature on the postoperative challenges confronted by pediatric inpatients was completed to identify the needs of patients and their parents after surgery and after hospital discharge. This brief report aims to answer specific questions relevant to 4 distinct themes: (1) Analgesic use and side effects: What are the common side effects of postoperative analgesics reported by patients? How do these side effects disrupt patients' quality of life? (2) Self-management strategies: What are the common self-care strategies that patients use to attenuate side effects of analgesics? (3) Education provided by healthcare providers: What education is provided by healthcare providers on analgesics and their side effects? What is the perception of patients and/or their parents on the education provided on analgesics and their side effects? (4) Patients' and parents' concerns: What are the concerns of patients and/or their parents in terms of medication use?

## 2. Methods

A literature search was conducted using the online databases of PubMed, Medline, and Scopus as well as using the snowballing method of reference tracking using combinations of the following terms: pediatric, analgesic, pain medication, side effects, adverse effects, nausea and vomiting, postoperative, postdischarge, self-care, self-management, management, self-care strategies, patient expectations, patient concerns, and education. Articles published between 1990 and 2019 were searched for. The year 1990 was chosen because this is when opioids were increasingly used and studied, and became the first line of treatment for pain.^12,20^

Studies included had to relate to postoperative analgesic use by pediatric patients. The literature search included peer-reviewed articles published in English and studies using pediatric patients or their parents as participants. The term parent refers to any adult responsible for the child's care and supervision. The search was also limited to studies investigating patients scheduled to undergo or who have undergone a surgery that required incision, anesthesia, and a postoperative hospital stay. Only studies investigating common medications used in North America were included. Articles that were excluded were those that investigated the postoperative period after 6 months, patients undergoing outpatient surgery, adult patients (mean age >18 years), and patients with cancer. These exclusions had the purpose of homogenizing the populations and narrowing the research being discussed.

Articles were screened and excluded based on the relevance of their abstracts and on whether they fulfilled the criteria by 2 independent reviewers. The full texts of the remaining articles were selected, read, and summarized by one reviewer.

## 3. Results

Ten studies met the criteria. A summary of the characteristics and results of the studies included are presented in Table 1.

**Table 1:** Study characteristics.

Article	# of participants	Patient age range (y)	Surgery type	Methodology	Results—side effects	Results—self-care strategies	Results—concerns	Results—interventions and education
Tesler et al.^25^	131 patients	8–17	Orthopedic, thoracic, abdominal, urologic, neurologic, other	Review of medical records Interviews on postoperative days 1–5	Patients prescribed, on average, 3.1 analgesics per child during 5 postoperative days Analgesics prescribed: acetaminophen with codeine, acetaminophen, meperidine, codeine, hydromorphone, hydrocodone with acetaminophen, aspirin Analgesics administered: morphine, meperidine, hydromorphone, codeine, acetaminophen with codeine, hydrocodone with acetaminophen, acetaminophen with aspirin	—	—	—
Iodice et al.^11^	54 patients	0–18	Cardiac	Retrospective review of patient records	Analgesics received: morphine, acetaminophen with NSAIDs (diclofenac or ibuprofen), acetaminophen alone Reported side effects: nausea, vomiting, itching	—	—	—
Howard et al.^9^	10, 079 patients	0–20	Head and neck, neurologic, plastic, cardiothoracic, urologic, orthopedic, general	Prospective data collection	Reported side effects: oversedation, respiratory depression, nausea or vomiting, itching	—	—	—
Swanson et al. (2012)	217 patients	0–12	Orthopedic	Retrospective review of patient charts	Most patients treated with nonopioids Medications: acetaminophen, morphine, codeine and Percocet Severe nausea and vomiting and sedation not observed	—	—	—
Chen et al.^3^	60 patients	7–14	ENT, orthopedic	RCT (hydromorphone vs morphine)	Analgesics: hydromorphone, morphine Reported side effects: nausea, vomiting, urinary retention No difference in side effects between both groups	—	—	—
Czarnecki et al.^5^	62 patients	10–19	Orthopedic	Retrospective review of charts including records of follow-up telephone interviews	Less side effects with oxycodone-CR than morphine Reported side effects: sedation, dizziness, constipation, pruritus, nausea, vomiting, respiratory depression PCA: sedation and pruritus Oxycodone-CR: constipation, nausea, dizziness	—	—	—
Lim et al.^14^	14 parents of patients	6–12	Not mentioned	Semistructured interviews	—	—	—	Parents' need to be involved in the child's postoperative care Parents' need for information and support from nurses
Sama et al.^17^	106 patients	0–15	Abdominal, orthopedic, urogenital, plastic, thoracic, neurologic, other	Prospective descriptive survey	Analgesics: acetaminophen, ketoprofen, tramadol, morphine Reported side effects: nausea, vomiting, urinary retention, respiratory depression	—	—	—
Simons and Roberson^18^	20 nurses and 20 parents of patients	Not mentioned	Moderate to major surgery	Matched interviews	—	—	Concerns about child's side effects and overconsumption of medication	Parents' need for more information on pain management Hesitancy approaching busy nurses Education approach not standardized Lack of communication between parents and nurses
Vincent et al.^27^	108 parents and their children	7–17	ENT, thoracic, orthopedic	Prospective, two-group, pretest-posttest, quasiexperimental study Educational intervention group vs standard education group	Analgesics: mostly acetaminophen	—	Concerns about efficacy of analgesics, the change in medication intake schedule after transition from hospital to home, side effects	No significant difference in parental satisfaction between both groups

ENT, ear, nose, throat; NSAIDs, nonsteroidal anti-inflammatory drugs; Oxycodone-CR, oxycodone—controlled release; RCT, randomized control trial.

### 3.1. Analgesic use and reported side effects

The time window observed in the included studies ranged from the day after surgery to a few days after discharge. Analgesics that were administered or prescribed to patients after surgery were acetaminophen,^11,17,24,25,27^ morphine,^3,5,11,17,24^ codeine,^24,25^ meperidine,^25^ hydromorphone,^3,25^ hydrocodone,^25^ oxycodone,^5^ tramadol,^17^ aspirin,^25^ diclofenac,^11^ ibuprofen,^11^ and ketoprofen.^17^ Tesler et al.^25^ found that the patients in their sample were prescribed 3.1 analgesics in the 5 days after surgery. Side effects reported by patients were respiratory depression,^5,9,17^ sedation,^5,9^ nausea,^3,5,9,11,17^ vomiting,^3,5,9,11,17^ pruritus,^5,9,11^ dizziness,^5^ constipation,^5^ and urinary retention.^3,17^ No literature was found on how these side effects may affect patients' quality of life.

### 3.2. Self-management strategies

Patients may engage in self-care activities to attenuate the severity and frequency of their side effects. However, no studies were found in which this was investigated in pediatric patients.

### 3.3. Education provided by healthcare providers

It was common for parents of patients to feel as though they are not adequately knowledgeable about pain management and potential side effects.^14,18^ Simons and Roberson^18^ found that, in the days after surgery, about half of the parents they interviewed reported the need for more information on pain management. Many parents reported feeling overwhelmed with the amount of information they had to take in about pain management and felt hesitant to approach busy nurses for more support.^14,18^ Parents identified the need for more involvement in the patient's recovery and the need for more support and education from nurses concerning analgesic use and side effects.^14,18^

### 3.4. Patients' and parents' concerns

Parents also have concerns about their child's analgesic use.^27^ Parents have expressed worries about the efficacy of analgesics, the change in medication intake schedule after transitioning from hospital to home, the overconsumption of medication, and the side effects of postoperative analgesics.^18,27^

## 4. Discussion

The results found made it difficult to address the specific queries of this review due to limited published research in the field. A major issue that came out of the review is that parents commonly report lacking knowledge about analgesic use and side effects. Pain management education should aim to provide parents and patients with adequate and consistent age-specific information about the possible side effects of postoperative drugs and their duration, and advise them on how to manage them.^6,15,28^ Although discussing pain management and side effects one-on-one with a healthcare provider is important, written information should be provided in addition to this.^10^ This combination would allow the patient and parents to not only discuss concerns with a healthcare provider but also look back at written information if forgotten. A mobile application containing information about the postoperative period may be useful for patients and allow for access at any time.^19^ The implementation of knowledge translation intervention programs such as Evidence-based Practice for Improving Quality, in which contextual program tailoring, leadership support, staff engagement, and time and resource allocation are important factors,^22^ has also been shown to improve patient pain outcomes in a hospital setting.^21,22^

This review highlighted that parents have many concerns about their child's medication use and side effects such as the efficacy of analgesics, the change in medication use after transitioning from hospital to home, the overconsumption of medication, and the side effects of analgesics.^18,27^ Patients and parents should be knowledgeable about analgesic use and the risk for side effects in the weeks before surgery and their concerns should continuously be addressed thereafter by the same team to avoid them forgetting information and to ensure correct and consistent education.^26^ Prescribing antiemetics based on the reason for symptoms and administering prophylactic treatment to patients is frequent.^4,28^ However, parents and patients may not be comfortable with the consumption of additional medications; therefore, clinicians should also educate patients on nonpharmacological strategies for pain management and side effects.

Furthermore, the fact that information given to patients and parents is being perceived as being inadequate may be due to a lack of research conducted on pediatric inpatients' perspectives on the challenges they experience postoperatively. Most studies investigating this topic have used pediatric patients who have undergone day-surgery. Although outpatients may be more at risk of feeling unknowledgeable after discharge, the invasiveness of inpatient surgery usually requires the consumption of more analgesics; therefore, they may differ in terms of postoperative experience. A systematic review including both populations should be conducted. Also, only 4 of the 10 studies reviewed used a qualitative approach for data collection.^5,14,18,25^ Additional studies using qualitative methods can allow us to gain a deeper understanding of the patients' perspective on which side effects they find most disruptive and how they manage them. Moreover, how daily living is affected and which self-care strategies are used are important to study because they may have a significant impact on recovery. Knowing which self-care activities patients engage in and whether they are effective is important so that they can potentially be recommended as alternative side effect management strategies.

## Disclosures

The authors have no conflicts of interest to declare.
